# Association between breast cancer risk factors and blood microbiome in patients with breast cancer

**DOI:** 10.1038/s41598-025-90180-3

**Published:** 2025-02-19

**Authors:** Jeongshin An, Hyungju Kwon, Se-Young Oh, Young Ju Kim

**Affiliations:** 1 Institute of Convergence Medicine Research, Ewha Womans University Mokdong Hospital, Ewha Womans University School of Medicine, 1071 Anyangcheon-Ro, Yangcheon-Gu, Seoul, 07985 Republic of Korea; 2 Department of Surgery, Ewha Womans University Mokdong Hospital, Ewha Womans University School of Medicine, 1071 Anyangcheon-Ro, Yangcheon-Gu, Seoul, 07985 Republic of Korea; 3 Department of Obstetrics and Gynecology, Ewha Medical Institute and College of Medicine, Ewha Womans University, Seoul, 07804 Republic of Korea

**Keywords:** Breast cancer, Fatty liver, Microbiome, Prognosis, Cancer, Microbiology, Health care, Risk factors

## Abstract

This study investigated the relationship between risk factors for breast cancer (BC) and the microbiome by comparing the microbiomes of BC patients with fatty liver disease to those with a normal liver. Bacterial extracellular vesicles were collected from each blood sample, and next-generation sequencing was performed. The analysis identified specific microbiome profiles shared among groups with hyperglycaemia, hyperlipidaemia, and high body mass index (BMI), which were then compared with functional biomarkers. In particular, the genus *Faecalibacterium* was a specific bacterium found in the groups with high concentrations of low-density lipoprotein cholesterol, high BMI, and fatty liver disease. Therefore, when the prognosis of patients with BC was analysed based on *Faecalibacterium* presence, it was confirmed that patients’ prognoses tended to deteriorate. In this study, BC risk factors, such as hyperglycaemia, hyperlipidaemia, fatty liver, and high BMI, were interconnected through the microbiome. This provides insights into how the risk factors for BC are linked and their impact on the microbiome and human health.

## Introduction

Breast cancer (BC) is the most common cancer in women worldwide, accounting for one in four female cancers^1^. The incidence of BC is closely related to dietary patterns, with the risk varying depending on whether individuals follow a Western or Mediterranean diet^2^. Dietary patterns significantly affect the microbiome^3^, and the microbiome composition can be used to predict the diagnosis and prognosis of BC^4,5^. Dietary habits play an important role in regulating the composition of gut microbiota^6^. Dysbiosis of the microbiome is associated with hyperglycaemia and hyperlipidaemia, increased body weight and body mass index (BMI), and the development of fatty liver disease^7^. Factors such as hyperglycaemia, hyperlipidaemia, obesity, and high BMI are closely linked to BC risk factors^8,9^.

Fatty liver disease is commonly diagnosed in BC patients undergoing endocrine therapy. The incidence of nonalcoholic fatty liver disease (NAFLD) tends to increase in patients with BC receiving aromatase inhibitors or selective oestrogen receptor modulators^10,11^. Additionally, a previous study demonstrated that fatty liver disease can affect patient prognosis^12^. In particular NAFLD, unrelated to endocrine therapy, increases the risk of death in patients with breast cancer^13^.

Risk factors including hyperglycaemia, hyperlipidaemia, and high BMI are shared between fatty liver disease and BC^9,14^. High fasting glucose levels are associated with BC proliferation and metastasis^15^, and hyperlipidaemia is a known risk factor for BC development^9^. Furthermore, the treatment of hyperlipidaemia with statins reduces BC recurrence and mortality^16^. This study analysed the relationship between the microbiome and the risk factors associated with both BC and fatty liver disease. Sequencing data were obtained by collecting bacterial extracellular vesicles (EVs) from the blood of patients with BC and was used to analyse the relationship between the microbiome and clinical data. This study aimed to identify specific bacterial genera that could potentially contribute to the development of fatty liver disease in patients with BC and potentially influence BC prognosis.

## Results

### Patient characteristics

**Fig. 1:**
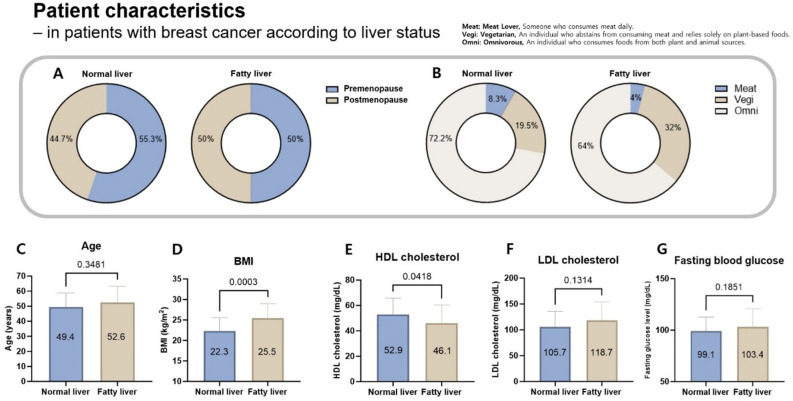
Characteristics of patients with BC separated into normal and fatty liver groups. Characteristics according to (**A**) menstrual status, (**B**) dietary pattern, (**C**) age, (**D**) BMI, (**E**) HDL cholesterol, (**F**) LDL cholesterol, and (**G**) fasting blood glucose.

This study includes 38 patients with BC and a normal liver (mean age, 49.4 years) and 27 patients with BC and a fatty liver (mean age, 52.5 years). Mann–Whitney U test results showed no significant difference between the two groups of age, suggesting that the two groups are similar (P = 0.3481) (Fig. 1). In patients with BC, 44.7% of those with a normal liver and 48.1% of those with a fatty liver were postmenopausal. Regarding dietary patterns, 27.8% of BC patients with a normal liver and 36% of BC patients with a fatty liver were found to have an unbalanced diet, such those high in meat or vegetable consumption. The proportion of patients on a balanced diet was 38.8% higher in the normal liver group than in the fatty liver group (Table 1). The difference in BMI between the fatty liver and normal liver groups was statistically significant according to the Mann–Whitney U test (P = 0.0003). The Mann–Whitney U test was also used for comparisons between other groups. Of the patients with a normal liver, 13.2% had low high-density lipoprotein (HDL) cholesterol (less than 40), whereas 29.6% of those with a fatty liver had low cholesterol, with a difference of 16.4% (P = 0.0481). In contrast, low-density lipoprotein (LDL) cholesterol and fasting glucose levels were not significantly different between the two groups (P = 0.1314, 0.1851).

**Table 1:** Patient characteristics.

	Breast cancer with normal liver	Breast cancer with fatty liver disease	*P* value
Female (total *N*)	38 (58.5%)	27 (41.5%)	
Age (year)	51.8 ± 10.1	51.5 ± 11.1	0.3481
Menopause status (*n*)			
Premenopausal	21 (55.3%)	14 (51.9%)	
Postmenopausal	17 (44.7%)	13 (48.1%)	
Dietary pattern (*n*)			
Meat lover	3 (7.9%)	1 (3.7%)	
Vegetarian	7 (18.4%)	8 (29.6%)	
Omnivore	26 (68.4%)	16 (29.6%)	
Unknown	2 (5.3%)	2 (7.4%)	
BMI (*n*)			
Mean (kg/m^2^)	22.3 ± 3.2	25.5 ± 3.5	0.0003
< 18.5	1 (2.6%)	1 (3.7%)	
18.6–24.9	32 (84.2%)	11 (40.7%)	
> 25	5 (13.2%)	15 (55.6%)	
HDL cholesterol (*n*)			
Mean (mg/dL)	52.9 ± 12.9	46.1 ± 14.2	0.0418
< 40	5 (13.2%)	8 (29.6%)	
≥ 40	32 (84.2%)	18 (66.7%)	
Unknown	1 (2.6%)	1 (3.7%)	
LDL cholesterol (*n*)			
Mean (mg/dL)	105.7 ± 30.1	118.7 ± 35.1	0.1314
< 130	29 (76.3%)	18 (66.7%)	
≥ 130	8 (22.1%)	8 (29.6%)	
Unknown	1 (2.6%)	1 (3.7%)	
Glucose			
Mean (mg/dL)	99.1 ± 13.7	103.4 ± 17.4	0.1851
< 99	23 (60.5%)	17 (63.0%)	
≥ 100	15 (39.5%)	10 (73.0%)	
Stage			
0	2 (5.3%)	1 (3.7%)	
I	21 (55.3%)	9 (33.3%)	
II	13 (34.2%)	13 (48.1%)	
III	2 (5.3%)	4 (14.8%)	

### Microbiome profiles in BC patients with the fatty and normal liver

**Fig. 2:**
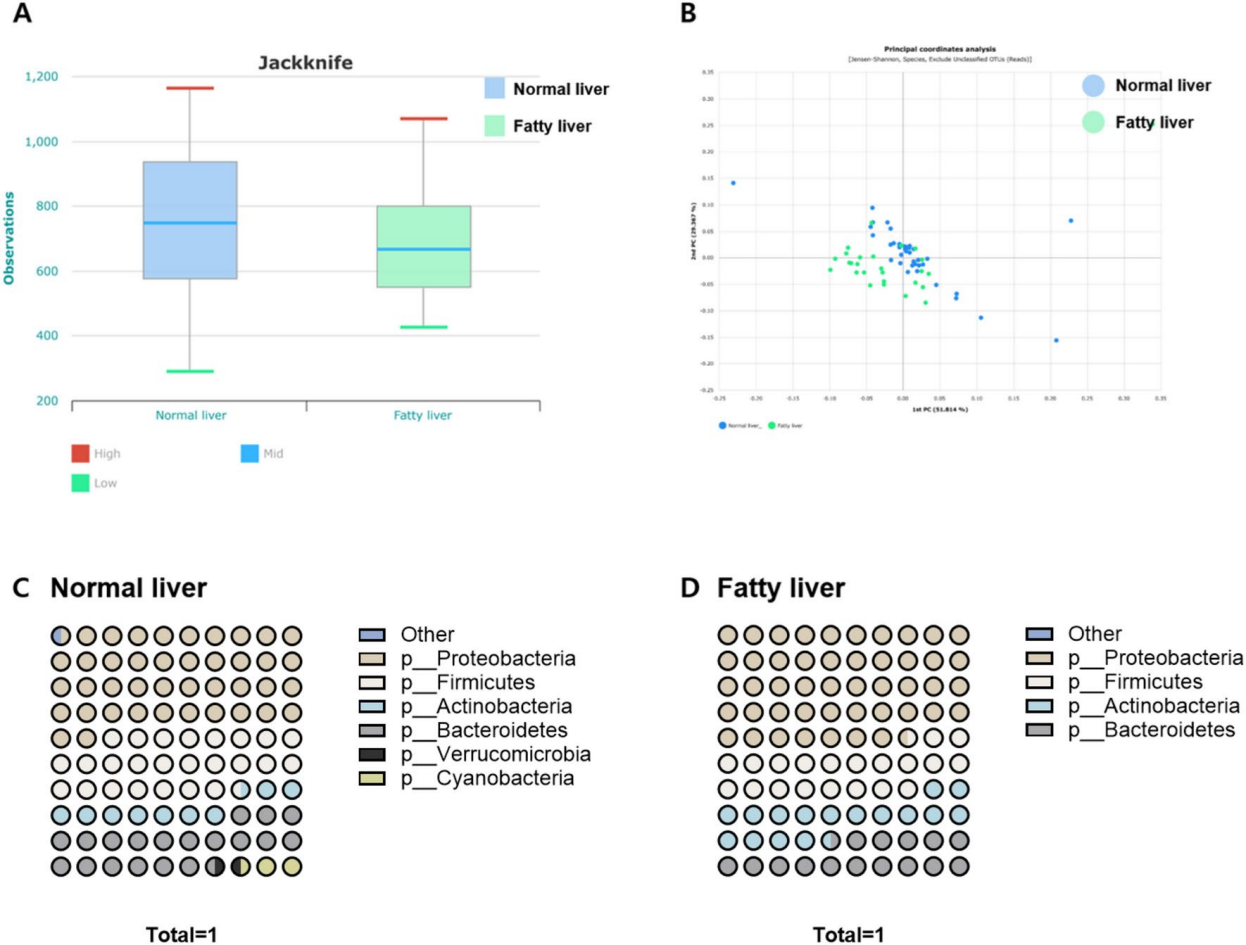
Serum microbiome profiles of normal and fatty liver groups in patients with BC. (**A**) α-diversity (Jackknife) and (**B**) β-diversity (Jensen-Shannon, species level) in normal and fatty liver groups. Microbiome profiles from patients with (**C**) normal and (**D**) fatty liver disease at the phyla level.

Among patients with BC, α and β diversity analyses were performed to compare the microbiota between the fatty and normal liver groups (Fig. 2). A jackknife analysis of α-diversity confirmed species richness, with the normal liver group showing a higher average value than the fatty liver group. However, the difference was not significantly different owing to the high variability observed in the normal liver group (Fig. 2A). β-diversity was evaluated using principal coordinates analyses. Although there were no significant differences between the two groups, differences observed at the species level are shown in Fig. 2B.

Microbiome composition was compared at the phylum level between the fatty and normal liver groups of patients with BC (Fig. 2C and D). Proteobacteria were the most abundant phyla in both groups, with a higher abundance in the fatty liver group than in the normal liver group. In contrast, Firmicutes and Bacteroidetes were more prevalent in the normal liver group, whereas Actinobacteria were more prevalent in the fatty liver group. The normal liver group had a greater diversity of phyla, including Verrucomicrobia and Cyanobacteria, which were nearly absent in the fatty liver group.

### Comparison of microbiome profiles according to fatty liver and related factors

**Fig. 3:**
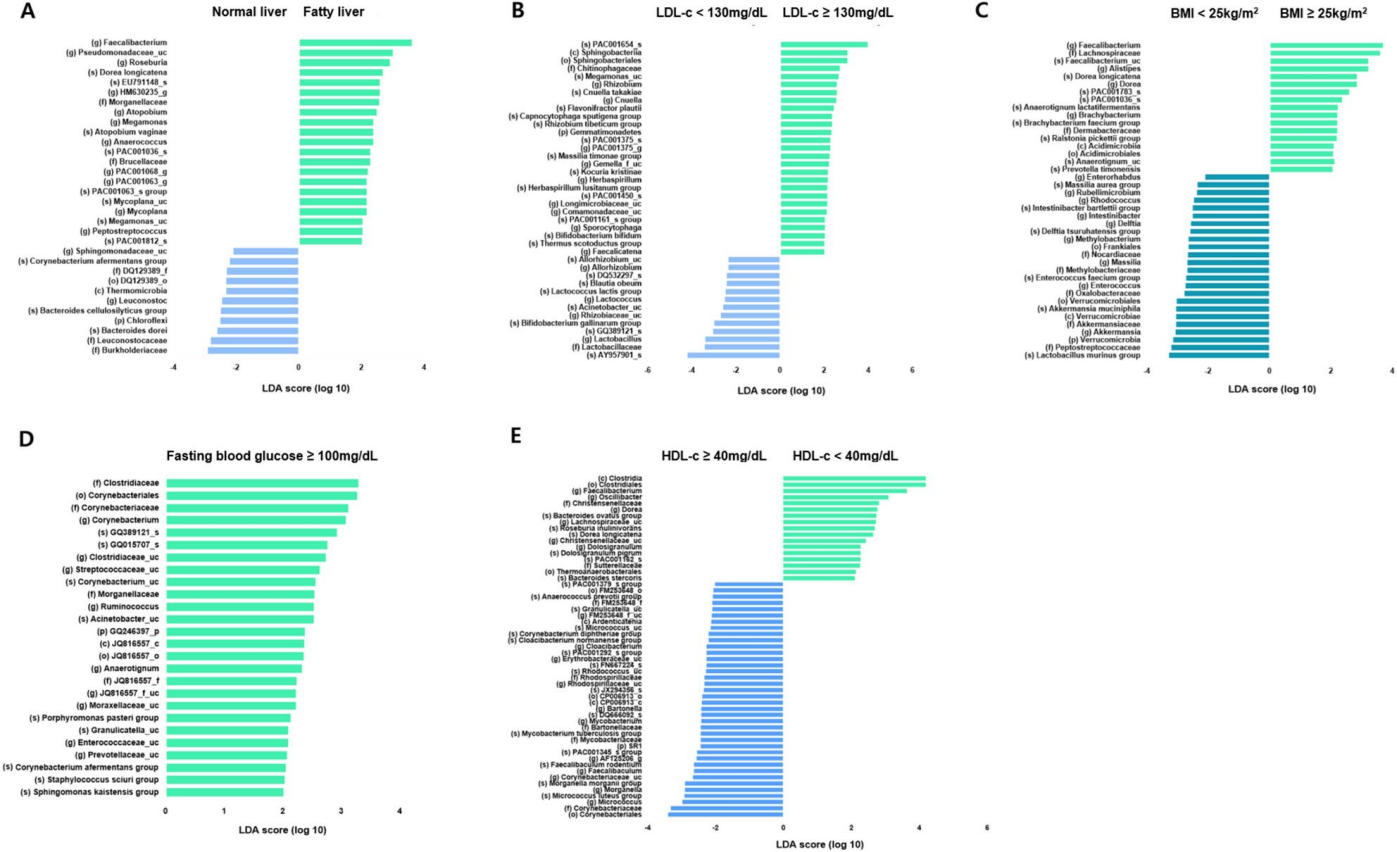
Significantly different taxa between each subgroup according to linear discriminant analysis effect size (LEfSe). The microbiomes specific to each group were analysed from phylum to species level by dividing the subjects into two groups according to (**A**) normal versus fatty liver disease, and by (**B**) LDL cholesterol (LDL-c), (**C**) BMI, (**D**) fasting blood glucose, and (**E**) HDL cholesterol (HDL-c) levels.

Patients with BC were divided into groups based on the presence of fatty liver disease and related components such as LDL and HDL cholesterol, BMI, and fasting blood sugar levels. This study aimed to identify specific bacteria associated with each of these factors. Linear discriminant analysis (LDA) scores were compared, focusing on bacteria with a P-value of less than 0.05. The LDA scores showed the specific bacteria, from phyla to species, significantly associated with each factor (Fig. 3). Figure 3A shows the differences in the microbiomes between the normal and fatty liver groups. The microbiome specific to fatty liver was predominantly genus *Faecalibacterium*, genus *Pseudomonadaceae_uc*, genus *Rosenuria*, and species *Dorea longicatena*. Figure 3B shows the results of the analysis focusing on LDL cholesterol levels. The patients with 130 mg/dL or higher LDL cholesterol were defined as high LDL group, while those with less than 130 mg/mL were designated as low LDL cholesterol group. In the high LDL cholesterol group, specific bacteria were identified in the following order: species *PAC001654_s,* class *Sphingobacteriia,* order *Sphingobacteriales,* family *Chitinophagaceae,* and species *Megamonas_uc*.

When comparing the high LDL cholesterol and fatty liver groups, *Megamonas_uc* was identified as a specific bacterium in both groups. Based on a BMI of 25, the subjects were divided into two groups: high BMI (over 25 kg/m^2^) and low BMI (under 25 kg/m^2^), and the differences between the two groups were analysed (Fig. 3C). In the high BMI group, the specific bacteria were the genus *Faecalibacterium*, family *Lachnospiraceae*, species *Faecalibacterium_uc,* genus *Alistipes*, and species *Dorea longicatena*. Among these, the *Faecalibacterium* and *Dorea longicatena* were found to be specific bacteria in both the high BMI and fatty liver groups. As shown in Fig. 3D, the patients were divided into two groups based on fasting blood glucose levels: above and below 100 mg/dL representing high and low glucose groups, respectively. Specific bacteria were found only in the high glucose group, and we attempted to identify the bacteria that overlapped between the high glucose group and the other groups. The high glucose and high BMI groups shared *Anaerotignum-uc* and *Anaerotignum lactatifermentans* as specific bacteria. The high LDL cholesterol and high glucose groups shared *Acinetobacter_uc*. The fatty liver and high glucose groups shared *Morganellaceae* and *Corynebacterium afermentans groups*.

When HDL cholesterol was divided into groups above and below 40 mg/dL, the genus *Dorea* was a common specific bacterium in both low HDL cholesterol and high BMI groups (Fig. 3E). The common specific bacteria among the fatty liver, low HDL cholesterol, and high BMI groups were *Faecalibacterium* and *Dorea longicatena*.

### Specific bacteria were common among BC risk factors

**Fig. 4:**
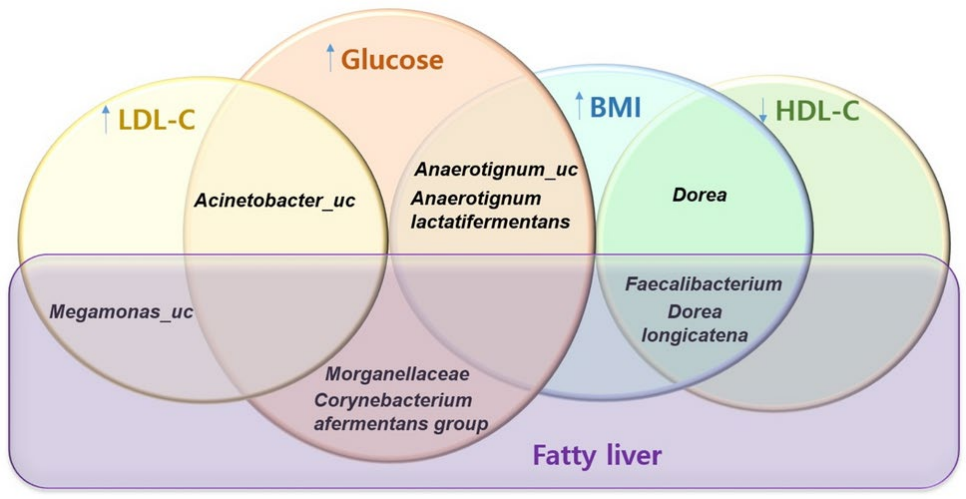
Venn diagram showing overlapping genera or species of bacteria in each subgroup from significantly different taxa according to linear discriminant analysis effect size (LEfSe).

Fatty liver is associated with high LDL cholesterol, high BMI, high glucose, and low HDL cholesterol levels. The relationships between these risk factors can be inferred from the bacteria that they share. Figure 4 summarises the results illustrated in Fig. 3.

The high-glucose group shared five specific bacteria with the high LDL cholesterol, high BMI, and fatty liver groups. Among these risk factors, high glucose group showed the greatest overlap in the bacteria. Notably, *Dorea longicatena* and *Faecalibacterium* were common specific bacteria shared by the fatty liver, high BMI, and low HDL cholesterol groups.

### The analysis of functional markers linked to associations between BC risk factors

**Fig. 5:**
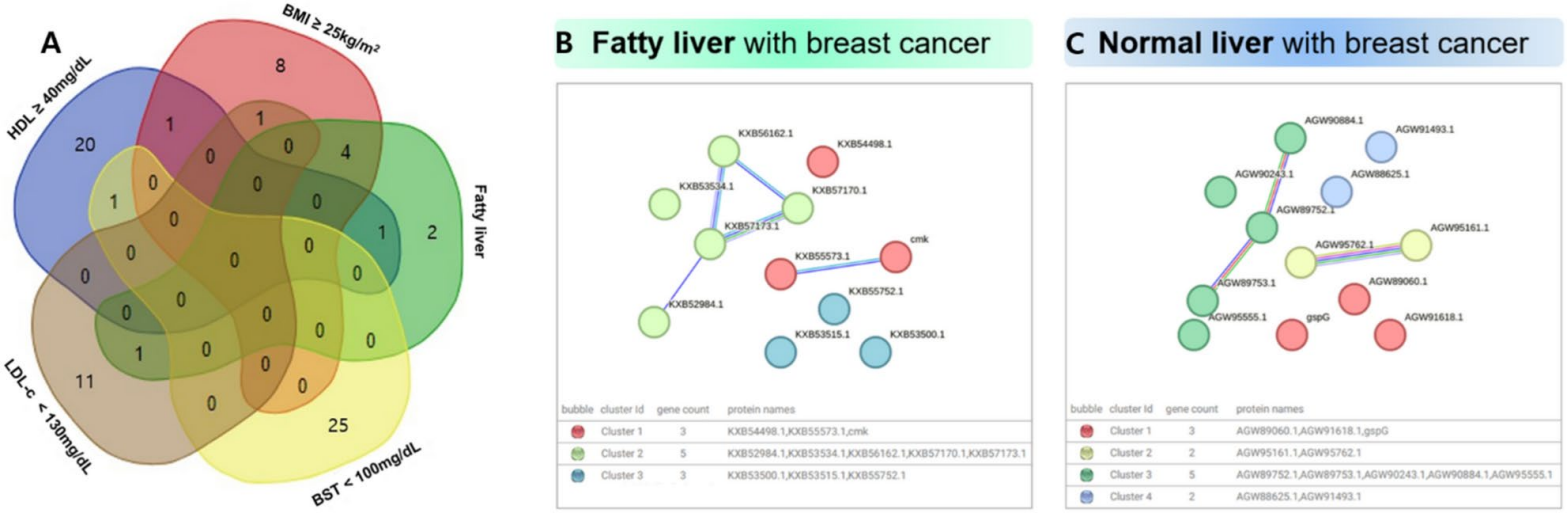
Analysis demonstrating the bacterial proteins specific to each group. (**A**) A Venn diagram illustrating the number of specific bacterial proteins in each subgroup, (**B**) Identifying the specific bacterial proteins in fatty liver disease and (**C**) normal liver and analysing the relationship between proteins in String.

To identify the specific protein markers in each group based on fatty liver condition, BMI, and HDL cholesterol, LDL cholesterol, and fasting glucose levels, specific proteins and pathways were determined for each group, and their overlaps are illustrated in a Venn diagram (Fig. 5A). Table 2 lists the proteins and pathways that overlapped between two or more groups. The overlapping functional biomarkers between the high BMI and fatty liver groups were CMP/dCMP kinase, DNA topoisomerase III, GntR family transcriptional regulator, and the DNA repair protein RadC. Putative transposases overlapped between the low BMI and normal LDL cholesterol groups. The antitoxin ParD1/3/4 biomarker was present in both the normal liver and LDL cholesterol groups (Table 2). Analysis based on a bacterial database revealed that proteins associated with fatty liver were involved in cellular processes related to DNA and RNA synthesis, replication, transcription, and repair. Bacterial proteins specifically associated with the normal liver were involved in activities and interactions related to DNA information exchange, positional changes, and metabolic processes (Fig. 5B and C).

**Table 2:**
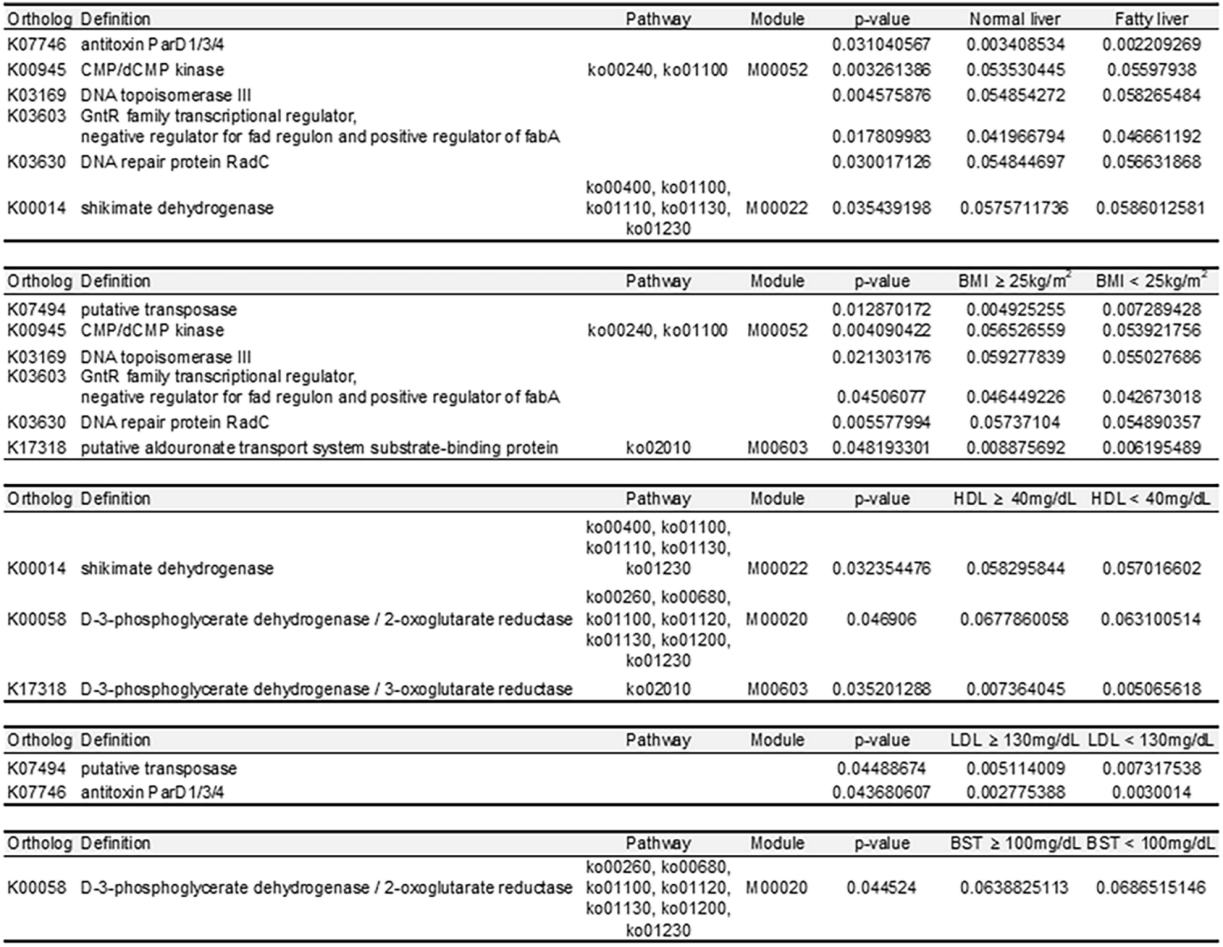
Functional biomarkers overlapping in each subgroup.

### Analysis of BC prognosis according to bacteria associated with BC risk factors

**Fig. 6:**
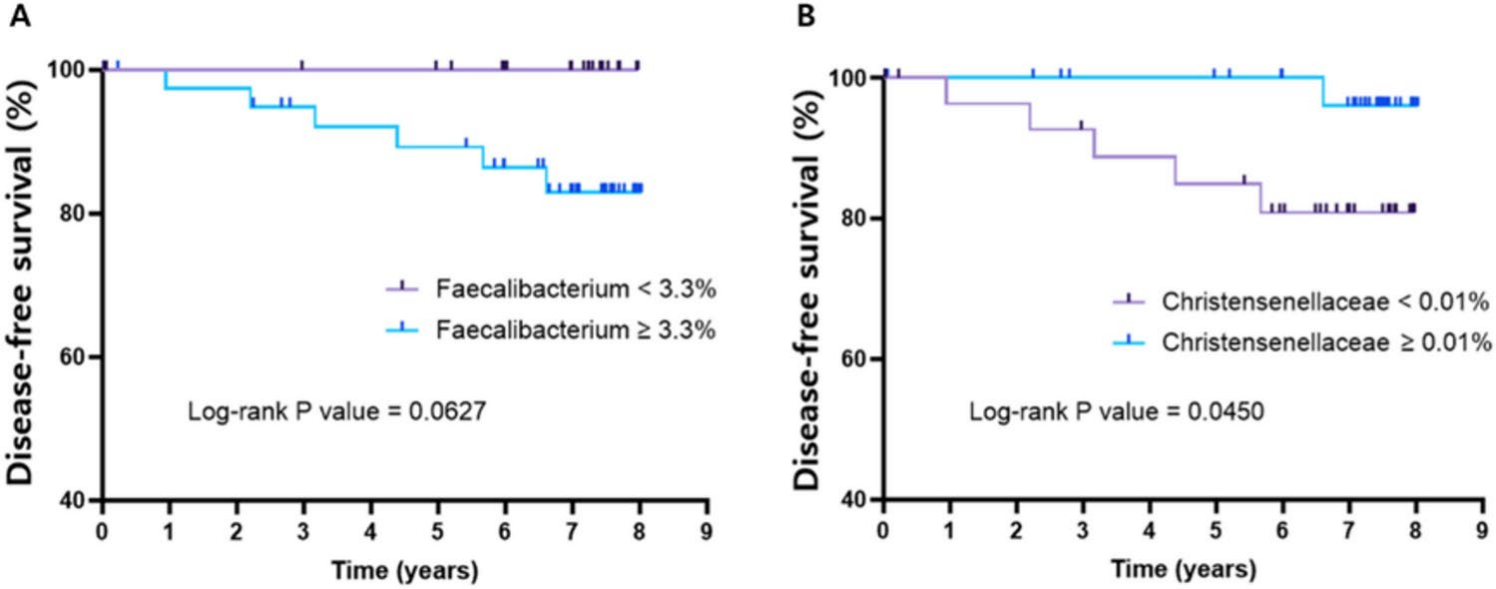
Analysis of disease-free survival by microbiome composition in relation to BC risk factors. The prognosis of patients with BC according to the abundance of (**A**) *Faecalibacterium* genus and (**B**) *Christensenellaceae* family was analysed using Kaplan–Meier analysis.

Based on the results shown in Fig. 5, the bacteria *Faecalibacterium* and *Dorea longicatena* overlapped across the following three risk factors: low HDL cholesterol, high BMI, and fatty liver. Thus, we identified bacteria associated with BC prognosis. *Faecalibacterium* was found to be correlated with prognosis (Fig. 6A). The optimal cut-off value for *Faecalibacterium* in patients with BC was 3.3%. The group with less than 3.3% *Faecalibacterium* exhibited a better prognosis (log-rank P = 0.0627). *Christensenellaceae*, which is inversely related to the BMI of the host^17^, was also evaluated for its association with BC prognosis. The optimal cut-off value for *Christensenellaceae* in patients with BC was 0.01%. Patients with *Christensenellaceae* levels above 0.01% showed better disease-free survival (log-rank P = 0.045) (Fig. 6B).

## Discussion

This study evaluated the association between microbiomes and BC risk factors, fasting glucose, LDL cholesterol, HDL cholesterol, and BMI, by analysing 65 blood samples from patients with BC to predict their prognoses. The fundamental distinction between previous blood microbiome studies^18^ and our current investigation resides in the methodology: instead of sequencing blood directly, we isolated and sequenced bacterial extracellular vesicles from the blood. This approach is fundamentally different from whole-genome sequencing using blood samples. Additionally, in the context of cancer development, chronic inflammation over prolonged periods is widely recognized as a contributing factor to the origins of cancer^19^. The relationship between disease and the microbiome is intrinsically interconnected, as disruptions in the balance of the microbiota can trigger inflammation, which in turn contributes to the development of various diseases, including breast cancer^20^. Like other diseases, breast cancer could also be influenced by the long-term inflammatory interactions with symbiotic microbiota^21^. Furthermore, bacterial products such as deoxycholic acid or butyrate, which translocate from the gut into the bloodstream via the portal vein could serve as evidence of this impact, highlighting the potential role of the blood microbiome in disease pathogenesis^22,23^.

By exploring the relationships among different BC risk factors, the potential contribution of these risk factors to the course of BC was assessed, and an overall schematic diagram illustrating the connection between each risk factor and microbiome was constructed (Fig. 4). The Venn diagram analysing the overlapping specific bacteria in each subgroup showed that high LDL cholesterol was associated with high glucose levels, which consequently related to high BMI. High BMI was associated with low HDL cholesterol levels. We also found that high LDL cholesterol and glucose levels were independently associated with fatty liver disease based on their microbiomes.

Interactions between hosts and bacteria, as well as between bacterial species, can occur through small molecules produced by these bacteria^24^. For example, the proteins produced by specific symbiotic bacteria destroy the cell walls of pathogenic bacteria and cause microecological changes^25^. Certain bacterial strains, such as *Lactobacillus Rhamnosus*, alter microecology by regulating the composition of the intestinal flora and restoring damaged intestinal barriers and host immunity^26^. Similarly, *Dorea*, which was identified as the common bacterial genus among the fatty liver, low HDL cholesterol, and high BMI groups in this study, is known for its ability to degrade polysaccharides, oligosaccharides, and sugars^27^. It has previously been reported to have a positive correlation with type 2 diabetes mellitus (T2DM)^28^. In our findings, *Dorea* was associated with high BMI and low HDL cholesterol, both recognized as risk factors for T2DM^29,30^. Previous research has suggested that *Dorea* links to immune regulation^31^. Specifically, in our study, *Dorea longicatena* was associated with high BMI, low HDL cholesterol, and fatty liver, sharing three key risk factors. These findings suggest that alterations in *Dorea* driven by these breast cancer risk factors may be associated with dysregulation of immune system. Our study identified *Megamonas* as the predominant bacteria shared among the individuals with high LDL cholesterol and fatty liver. *Megamonas* has been identified in previous studies as a dominant gut microbial species in patients with non-metastatic breast cancer, with its deficiency linked to bone metastasis^32^. Considering the role of the microbiome in lipid transport, metabolism, and steroid hormone biosynthesis—which are critical in breast cancer development and bone metastasis—these bacteria may influence these processes^32^. *Corynebacterium* was associated with diacylglycerol, a lipid second messenger involved in intracellular signal transduction^33^. In our study, *Corynebacterium* was linked to high glucose levels and fatty liver, suggesting a plausible connection to fatty liver via lipid second messenger pathways. Diacylglycerol kinase alpha (DGKα) is a known proliferation factor in cancer cells^34^, and disruptions in the activity and abundance of DAG effectors have been associated with tumor progression and metastasis^35^. Consequently, the positive correlation between *Corynebacterium* and DAG suggests a potential link between *Corynebacterium* and the pathogenesis of breast cancer.

Microecological changes in the human body have been linked to chronic diseases, such as breast and liver disease^36,37^. Bacterial proteins in the microbiome associated with risk of BC may be involved in these microecological changes (Supplemental Table 1). Proteins that are particularly important in BC are expected to overlap with diverse risk factors. CMP/dCMP kinase, DNA topoisomerase III, GntR family transcriptional regulator, and RadC were specific bacterial proteins in patients with fatty liver disease and with a high BMI. These bacterial proteins are mainly involved in cellular nucleic acid synthesis, DNA replication, and DNA transcription^38–40^, all of which are related to DNA repair mechanisms. The pathological progression of NAFLD involves fatty acid degeneration, lipotoxicity, and inflammation. Lipotoxicity induces oxidative stress, which causes mitochondrial dysfunction and the generation of reactive oxygen species, eventually leading to liver cell death^41^. In response to a microenvironment damaged by oxidative stress, symbiotic bacteria could release bacterial proteins involved in DNA repair mechanisms to restore normal microenvironment.

The low LDL cholesterol group of patients with BC shared a putative transposase as a specific common protein with the group of patients with a low BMI, while the antitoxin ParD1/3/4 was identified as a specific protein shared with the normal liver group. Putative transposases assist during gene transfer, regulate gene expression, and create new genetic traits^42^, and the antitoxin ParD1/3/4 which neutralises bacterial toxins, is essential for cell survival^43^. These findings suggest that bacterial proteins in the patients with low LDL cholesterol and normal BMI and liver groups support the microenvironment maintaining cell survival, such as genetic polymorphisms and antitoxins, rather than compensating for cell damage.

When the host environment deteriorates in fatty liver disease in patients with a high BMI, it can be interpreted that not only the host but also the symbiotic bacteria are attempting to recover the normal microenvironment. *Faecalibacterium* shared among groups with high LDL cholesterol, high BMI, and fatty liver disease, is highly expressed in patients with poor BC prognosis. This result is contradictory to that of the previous study, where *Faecalibacterium spp.* was found to be low in stool samples from patients with existing fatty liver disease and obesity^44^. This difference is likely due to the different donor groups and sample types used between this and the prior study. Another previous study analysed the microbiome from the stool samples of patients without BC, whereas our study examined the microbiome from the blood samples of patients with BC. Since there has been no study of microbiomes comparing risk factors based on blood samples of patients with BC to date, the results of this study provide valuable insights into how the microbiome in the blood may be associated with BC risk. Based on our findings, it can be concluded that *Faecalibacterium* is associated with BC risk factors and poor BC prognosis. Some *Faecalibacterium spp.,* such as *Faecalibacterium prausnitzii*, are involved in regulating inflammation and the immune system^45^. When comparing the expression patterns of bacterial proteins, it can be inferred that bacterial proteins involved in DNA repair are highly expressed to compensate for cell damage. The expression of bacterial proteins in the microbiome varies depending on the specific conditions or needs of the host.

A limitation of this study is its small sample size. Patients with BC and liver abnormalities other than fatty liver disease were excluded, and the control group was limited to patients with BC without liver abnormalities, including fatty liver disease. This exclusion inevitably narrowed the sample size of this study. However, this study presents a novel perspective on the relationship between risk factors in patients with BC and the microbiome. The microenvironment plays an important role in regulating cancer development, influencing the progression of BC and various other cancers. According to this study, bacterial proteins derived from microbiome may contribute to the host microenvironment.

Microbiomes associated with specific risk factors produce bacterial products that adapt to each environment. These microbiomes are expected to create a microenvironment that supports host cell functions.

## Materials and methods

### Enrolment of patients

Those who were diagnosed with BC and did not undergo surgery or treatment, and those who consented to participate in the study were included. Liver disease was classified using liver ultrasonography or abdominal computed tomography. This study divided patients with BC according to their liver status into those with normal liver and those with liver disease, and among those with liver disease, fatty liver and other liver diseases were classified. Patients with BC with liver masses, gallbladder polyps, or gallbladder stones and those with liver disease other than fatty liver disease were excluded (n = 32). The participants were divided into patients with BC and normal livers (n = 38) and those with fatty liver disease (n = 27). Individuals with a history of taking drugs or supplements, such as antibiotics or probiotics, within one month before blood collection were excluded from the study. Informed consent was obtained from all study participants. This study was approved by the Institutional Review Board of Ewha Womans University Mokdong Hospital (approval number: EUMC 2014–10-005–019). All methods were performed in accordance with the relevant guidelines. Patients with stage 0–3 BC were recruited and monitored for metastasis and recurrence.

### Bacterial EV isolation and next-generation sequencing

Bacterial EVs were collected from the patient’s blood using a method described previously^46^. Briefly, blood samples were collected in serum-separating tubes, and serum was separated. Blood was centrifuged at 1500 × *g* for 15 min at 4 °C and diluted with 1 × phosphate-buffered saline (PBS pH 7.4, ML008-01; Welgene, Gyeongsan, Korea). The supernatant was obtained by centrifugation at 10,000 × *g* and 4 °C for 1 min. The supernatant was filtered using a 0.22 μm filter and ultracentrifuged at 150,000 × *g* for 3 h at 4 °C using a 45 Ti rotor (Beckman Instruments, Brea, CA, USA). The resulting pellet containing EVs was diluted in PBS and stored at -80 °C. DNA was isolated using an isolation kit (MoBio PowerSoil DNA Isolation Kit; Qiagen, Hilden, Germany). The extracted DNA was quantified using a QIAxpert system (Qiagen). Amplification was performed using V3 forward and V4 reverse primers targeting the V3-V4 hypervariable region of *16S* rRNA. Primer sequences were 16S_V3_F (5'-TCGTCGGCAGCGTCAGATGTGTATAAGAGACAGCCTACGGGNGGCWGCAG-3') and 16S_V4_R (5'-GTCTCGTGGGCTCGGAGATGTATAAGAGACAGGAC-TACHVGGGTATCTAATCC-3')^47^. A library was prepared using the PCR product, and next-generation sequencing was performed using MiSeq (Illumina, San Diego, USA).

### Microbiome analysis

In this study, The EzBioCloud platform (database updated on August 23, 2023) was used to compare the characteristics of each subgroup and identify genetic and functional markers^48^. Species-level classification was performed using Average Nucleotide Identity (ANI) calculations, requiring ANI ≥ 95% and coverage ≥ 20%. If these criteria were not met, additional classification was conducted using 16S rRNA sequences (> 1300 bp). The EzBioCloud 16S rRNA database was integrated with QIIME 2 (version 2024.10) and Mothur (version 1.48.0) pipelines for OTU (Operational Taxonomic Unit)-based classification. Processes such as OTU selection, chimera sequence removal, and sequence alignment were applied to ensure accurate classification. Species abundance was analysed and normalized based on metagenome data, utilizing EzBioCloud’s comprehensive genome database to ensure high accuracy and reproducibility. Microbiome data were analysed using GraphPad Prism software (version 10.2.3). All microbiome data were analysed only for data with P-values < 0.05, and LDA scores were analysed only for data with absolute values > 2. Analyses of protein–protein interactions and functional analyses were performed using String software (version 12).

### Analysis of clinical data from patients with BC

This study collected clinical data on eating habits, menopausal status, LDL cholesterol, HDL cholesterol, fasting blood sugar levels, and BMI from patients with BC. In terms of eating habits, people who ate meat at every meal were designated “meat lovers”, those who do not eat meat and ate only vegetarian food were considered vegetarians, and those who ate a balanced diet were considered omnivores. According to the World Health Organization criteria, BMI was classified as low BMI if it was below 18.5 kg/m^2^, normal BMI as between 18.6 and 24.9 kg/m^2^, and high BMI as above 25 kg/m^2^. For convenience, BMI was divided into high and low, and a BMI of 25 kg/m^2^ was used as the cut-off^49^. HDL was classified as low HDL if it was below 40 mg/dL, and high was classified as 40 mg/dL or above^50^. LDL cholesterol was classified as high if it was > 130 mg/dL and low if it was less than 130 mg/dL^51^. Fasting blood sugar was divided into a low glucose group if it was less than 100 and a high glucose group if it was > 100^52^. The patients’ stages ranged from stage 0–3, and patients at stage 4 at the time of diagnosis were excluded.

## Supplementary Information

Supplementary Information 1.

## Data Availability

The raw and processed metagenome sequencing data can be accessed via the Sequence Read Archive under the BioProject ID: PRJNA834582.
