# Supplementary figures and images for: Association between breast cancer risk factors and blood microbiome in patients with breast cancer
Source: Sci Rep. 2025 Feb 19;15:6115. doi: 10.1038/s41598-025-90180-3 (PMC11840066; doi:10.1038/s41598-025-90180-3)

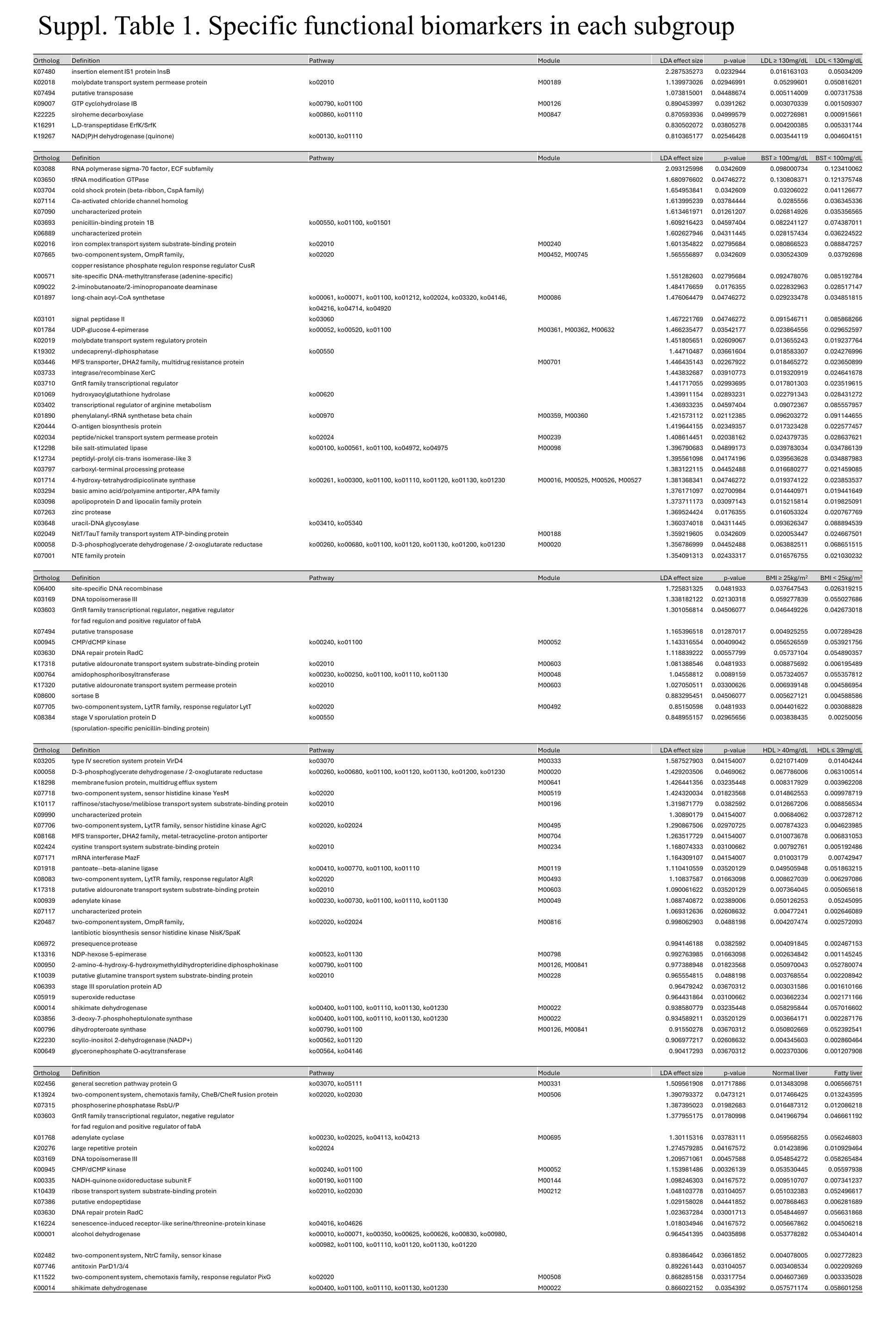

Supplement: Supplementary file 1 — Supplementary Information 1. [file 41598_2025_90180_MOESM1_ESM.tif]
